# Evaluating the Quality of Selected Commercial Probiotic Products, Both Dietary Supplements and Foods for Special Medical Purposes

**DOI:** 10.3390/foods15020373

**Published:** 2026-01-20

**Authors:** Anna Zawistowska-Rojek, Justyna Rybak, Paulina Smoleń, Agnieszka Kociszewska, Paweł Rudnicki-Velasquez, Karolina Węgrzyńska, Tomasz Zaręba, Stefan Tyski, Anna Baraniak

**Affiliations:** 1 Department of Pharmaceutical Microbiology and Laboratory Diagnostics, National Medicines Institute, Chełmska 30/34, 00-725 Warsaw, Poland; j.rybak@nil.gov.pl (J.R.); p.smolen@nil.gov.pl (P.S.); a.kociszewska@nil.gov.pl (A.K.); k.wegrzynska@nil.gov.pl (K.W.); t.zareba@nil.gov.pl (T.Z.); s.tyski@nil.gov.pl (S.T.); 2 Department of Falsified Medicines and Medical Devices, National Medicines Institute, Chełmska 30/34, 00-725 Warsaw, Poland; p.rudnicki@nil.gov.pl

**Keywords:** dietary supplements, probiotics, product quality, viable cell count, flow cytometry, MALDI-TOF MS, qPCR, microbiological purity

## Abstract

Probiotics are live microorganisms that provide health benefits when administered in adequate amounts. Due to the increasing popularity of probiotic supplements, concerns have arisen regarding their quality, microbial composition, and safety. This study aimed to evaluate the quantitative and qualitative characteristics of the selected probiotics available on the Polish market, including both dietary supplements and foods for special medical purposes, and to compare the obtained results with the information provided on the product labels. Fifteen commercial probiotic products were analysed. Viable microorganism counts were determined using the traditional culture-based plate count method and by flow cytometry for selected products. Species identification was performed using MALDI-TOF MS and qPCR, whereas microbiological purity testing was conducted to confirm the absence of pathogenic bacteria. Significant differences were observed between the declared and experimentally determined numbers of viable microorganisms. Only a few products maintained bacterial counts consistent with label claims, while most contained considerably low viable cells. Flow cytometry revealed higher viable cell counts than plate counting, indicating the presence of viable but non-culturable bacteria. The declared species composition of the strains was mostly confirmed, although in several cases, undeclared probiotic microorganisms were identified. All tested products were free from pathogens. The study indicates significant discrepancies in the quality of probiotic supplements available on the Polish market. From a consumer perspective, these findings highlight the importance of verifying probiotic quality and suggest that not all commercial products may guarantee the full range of claimed health benefits. The implementation of standardised analytical procedures and enhanced quality control measures is therefore essential to ensure the product safety, strain authenticity, and reliability of health-related claims.

## 1. Introduction

Microorganisms referred to as probiotics are administered to support the body’s physiological functions and may also reduce the risk of many diseases, shorten their duration, or alleviate symptoms. They are widely incorporated into functional foods and dietary supplements and are primarily selected based on their safety profile, stability, and documented effects. Probiotic products commonly contain bacterial strains from the family *Lactobacillaceae* (*Lacticaseibacillus rhamnosus*, *Lacticaseibacillus casei*, *Lacticaseibacillus paracasei*, *Lactobacillus acidophilus*, *Lactiplantibacillus plantarum*, *Limosilactobacillus fermentum*, *Limosilactobacillus reuteri*, and *Lactobacillus delbrueckii* ssp. *bulgaricus*) and the genus *Bifidobacterium* (*B. infantis*, *B. animalis*, *B. bifidum*, and *B. longum*), as well as the yeast *Saccharomyces cerevisiae* var. *boulardii* [1,2,3,4].

The effects of probiotics are multifactorial and may involve both local actions within the gastrointestinal tract and systemic effects throughout the body. Their main mechanisms of activity include modulation of the intestinal microbiota, stimulation of the immune system, enhancement of the intestinal epithelial barrier, and production of antimicrobial substances such as organic acids, bacteriocins, hydrogen peroxide, and short-chain fatty acids [4,5,6]. Furthermore, probiotics contribute to the synthesis of vitamins, mainly from the B group and vitamin K, and can improve the absorption of essential minerals such as calcium, iron, zinc, and copper [4,7]. All of this means that they are not only useful in maintaining gut microbial balance but may also play an important role in the prevention and adjuvant treatment of various diseases. Documented benefits include the alleviation of symptoms in irritable bowel syndrome (IBS), inflammatory bowel disease, allergies, atopic dermatitis (AD), diabetes, obesity, and mood disorders [7,8,9,10]. In the context of the gut–brain axis, particular attention has been given to so-called psychobiotics, a group of probiotic microorganisms capable of influencing neurotransmitter levels and nervous system function, which may be useful in the treatment of depression and anxiety [11].

Due to growing consumer interest, probiotic formulations containing single or mixed cultures of live microorganisms have become widely available on the market in various forms, such as foods, capsules, suspensions, sachets, granules, tablets, and aerosols [12,13]. However, the therapeutic efficacy of probiotics largely depends on the quality and stability of the consumed product. Since each probiotic strain exhibits unique biological properties, accurate identification and maintenance of the viable cell counts until the end of shelf life are crucial [14]. Probiotic preparations should meet several essential criteria, such as accurate and complete strain designation, documented safety, at least one clinical study confirming efficacy, and verified presence of viable microorganisms throughout the declared shelf life [3]. In practice, these requirements are not always fulfilled. Numerous quality control studies of commercial products have revealed inconsistencies between declared and actual cell counts, the presence of undeclared strains, or even microbiological contamination [12,14,15,16].

One of the key challenges for probiotic quality remains ensuring their stability during production, storage, and passage through the gastrointestinal tract. Numerous environmental factors including temperature, water activity, oxygen exposure, and UV radiation also contribute to the reduction in cell viability [13,17]. In response to these issues, a variety of protective technologies has been developed, and among them are microencapsulation methods using biocompatible polymers such as alginates, chitosan, cellulose, and whey proteins. These approaches enhance bacterial survival under harsh environmental conditions and enable controlled release in the intestine [18,19,20].

The assessment of probiotic product quality includes both the quantitative and qualitative determination of probiotic organisms as well as the verification of microbiological purity. The traditional and most widely used approach for quantifying viable cells is the culture method, based on counting colony-forming units (CFUs). Although this technique is reliable, it remains labour-intensive, time-consuming, and unable to detect viable but non-culturable (VBNC) cells that stay metabolically active without forming colonies on solid media [21,22,23]. Therefore, flow cytometry, as recommended by ISO 19344:2015 [24], is increasingly applied as a rapid and precise method for determining the number of viable and non-viable bacterial cells using fluorescent dyes [21,25]. This method also enables the detection of VBNC populations, making it a valuable tool for evaluating probiotic viability.

The species identification of strains can be performed using both classical phenotypic methods and advanced instrumental or molecular techniques. Biochemical tests are simple and cost-effective, but their accuracy is limited, especially for closely related species [21]. In turn, matrix-assisted laser desorption/ionisation time-of-flight mass spectrometry (MALDI-TOF MS) allows for rapid and relatively inexpensive species identification based on cellular protein profiles, though its reliability depends on the quality of spectral reference databases and may be limited for certain *Lacticaseibacillus* spp. [26,27]. Complementary molecular methods using polymerase chain reaction (PCR) and real-time quantitative PCR (qPCR) are often employed to confirm and refine MALDI-TOF MS results, enabling the sensitive detection and identification of probiotic strains even in multi-strain formulations [22,26].

An equally crucial aspect of probiotic product evaluation is microbiological purity. Probiotics should be free from pathogenic bacteria such as *Escherichia coli*, *Salmonella* spp., *Staphylococcus aureus*, and *Listeria monocytogenes*, in accordance with the requirements of pharmacopoeias and regulatory bodies such as the Food and Drug Administration and the European Food Safety Authority [21,28]. The presence of contaminants may not only pose a health risk but can also affect the efficacy of the preparation by competing with probiotic strains or altering their metabolic activity. Therefore, microbiological purity is essential to ensure the safety, effectiveness, and reproducibility of probiotic action [16,28].

The aim of the present study was to perform a comprehensive evaluation of the quality of selected probiotic products available on the Polish market, including both dietary supplements and foods for special medical purposes. The analysed products were intended for various health applications, such as AD, IBS, allergies, diarrhoea, and abdominal pain, as well as psychobiotics supporting the nervous system. The study involved determining the number of viable probiotic microorganisms using two analytical methods, the culture-based method and flow cytometry, in order to compare their suitability for assessing viability. In addition, qualitative composition and strain identification were performed using MALDI-TOF MS and qPCR. Finally, the microbiological purity of the tested products was evaluated to confirm the absence of pathogenic microorganisms and to assess product safety.

## 2. Materials and Methods

### 2.1. Product Characteristics

Fifteen probiotic products available on the Polish market (all those offered by pharmacies; Table 1) were selected for analysis, including both dietary supplements (DSs) and foods for special medical purposes (FSMPs).

The tested products comprised single-strain (five products) and multi-strain (ten products) formulations containing bacteria from the *Lactobacillaceae* family (*L. rhamnosus*, *L. plantarum*, *L. acidophilus*, *L. helveticus*, *L. paracasei*, *L. casei*, *L. delbrueckii* subsp. *bulgaricus*, and *L. reuteri*), *Bifidobacterium* spp. (*B. breve*, *B. longum*, *B. animalis*, *B. bifidum*, *B. infantis*, and *B. lactis*) and *Bacillus* spp. (*B. coagulans*), and *Saccharomyces cerevisiae* var. *boulardii*. All products were stored according to the manufacturer’s recommendations and were analysed before the expiration date. The study involved determining the number of viable microorganisms in each item, microorganism identification, and an assessment of microbiological purity.

### 2.2. Determination of Microorganism Count

#### 2.2.1. Pour Plate Method

The viable microorganism count was determined using the pour plate method, following the procedure described by Zawistowska-Rojek et al. [21]. The test was performed twice, once for all examined products and once again for those selected for flow cytometry analyses. A weighed sample was dissolved in a buffered NaCl–peptone solution at pH = 7 (Graso Biotech, Starogard Gdański, Poland) and homogenised. A series of tenfold serial dilutions was prepared, and 1 mL from each dilution was transferred, in duplicate, onto Petri dishes and overlaid with an appropriate growth medium depending on the type of microorganism. The number of viable cells was calculated per product dose (CFU/dosage form). All analyses were performed in parallel replicates.

For bacteria belonging to the family *Lactobacillaceae*, DeMan Rogosa and Sharpe Agar, MRS Agar (Merck-Millipore, Darmstadt, Germany), was used (37 °C ± 1 °C, 72 h atmosphere supplemented with CO_2_). Bacteria of the genus *Bifidobacterium* were cultured on Bifidobacteria Selective Medium, BSM agar (Merck-Millipore, Darmstadt, Germany), with mupirocin (Merck-Millipore, Darmstadt, Germany) under anaerobic conditions (37 °C ± 1 °C, 72 h). Yeasts were cultured on Sabouraud Dextrose Agar supplemented with chloramphenicol (Biomaxima, Lublin, Poland) under aerobic conditions (37 °C ± 1 °C, 72 h) [21].

#### 2.2.2. Flow Cytometry

The flow cytometry analysis was conducted at a different time point, and due to expiration date constraints, only seven of the fifteen tested probiotic products could be used. These products were assessed for the number of viable and non-viable bacterial cells by flow cytometry using a BD FACSCanto II device (BD Biosciences, Franklin Lakes, NJ, USA). Prior to analysing each probiotic product, the instrument was calibrated using BD CS&T quality control beads (BD Biosciences, Franklin Lakes, NJ, USA). The tested samples originated from the same preparation as those employed in the repeated pour plate method, and their further processing for flow cytometry testing was in accordance with Protocol A of the ISO 19344:2015 [25]. The used fluorescent dyes, 5-(and-6)-carboxyfluorescein diacetate, cFDA (permeant, green dye) and propidium iodide, and PI (non-permeant, red dye) were procured from Invitrogen (Waltham, MA, USA).

The data were analysed with FACSDiva software (v8.0.1, BD Biosciences, Franklin Lakes, NJ, USA). A blank control and individually stained cFDA and PI controls were prepared to separate bacterial events from the background noise on a multiparameter dot plot cytogram. The *x*-axis of the cytogram indicates the logarithmic green fluorescence intensity of cFDA (viable bacteria cells). The *y*-axis indicates the logarithmic red fluorescence of PI (non-viable bacteria cells). The number of viable bacteria cells was expressed as active fluorescence units (AFUs) per dosage form (AFU/dosage form), while the count of non-viable bacteria cells was expressed as nonactive fluorescence units (N-AFUs) per dosage form (N-AFU/dosage form).

### 2.3. Strain Identification

#### 2.3.1. MALDI-TOF MS

One sample from each of the different morphological colonies of microorganisms obtained by the pour plate method for all tested products was identified using MALDI-TOF MS (Bruker Daltonics GmbH & Co. KG, Bremen, Germany). Protein spectra generated for each isolate were compared against reference databases. The degree of similarity between the mass spectrum of the tested isolate and the reference spectrum was assessed using a scoring system. A score value (SV) ≥ 2.00 was interpreted as a highly probable identification at the species level, while SVs ranging from 1.70 to 1.99 were considered indicative of identification at the genus level. An SV below 1.70 was regarded as unreliable or having limited similarity to reference strains available in the database.

#### 2.3.2. Nucleic Acid Isolation and qPCR

Bacterial DNA was extracted from all study products (Table 1). Prior to isolation of genetic material, the powder samples were pre-incubated at 37 °C for 3 h in MRS broth (Merck-Millipore, Darmstadt, Germany). Then, nucleic acids were eluted using the Lab-Aid 824s Nucleic Acid Extraction System (Zeesan Biotech, Xiamen, China) with the Lab-Aid 824s DNA Extraction kit (Zeesan Biotech, Xiamen, China) according to the manufacturer’s instructions. DNA concentrations were normalised to 70 ng/µL after determining their strength and purity by a NanoDrop 2000 spectrophotometer (Thermo Fisher Scientific, Waltham, MA, USA).

The detection of six strains from the *Lactobacillaceae* family and five *Bifidobacterium* spp. was conducted using specific primers, as described previously (Table 2).

All qPCR reactions were performed for primer pairs identifying a single bacterial species using a CFX96/384 TouchTM device (Bio–Rad, Hercules, CA, USA) with Maxima SYBR Green/ROX qPCR Master Mix (2X) (Thermo Fisher Scientific, Waltham, MA, USA) according to the programme recommended by the test manufacturer. All products were tested for the presence/absence of each of the listed bacterial species. The DNA isolated from *B. animalis* subsp. *lactis* DSM 10140, *B. longum* subsp. *longum* DSM 20219, and *L. helveticus* DSM 3748 (German Collection of Microorganisms, Braunschweig, Germany), as well as *L. rhamnosus* ATCC 53103, *L. paracasei* ATCC 334, *L. acidophilus* ATCC 4356, *L. plantarum* ATCC 14917, *B. bifidum* ATCC 11863, *B. breve* ATCC 15700 (American Type Culture Collection, Manassas, VA, USA), and two genetically characterised isolates from our own collection, *L. reuteri* and *B. infantis*, was used as a positive control in corresponding separate qPCR reactions. Results were interpreted as positive based on the cycle quantification (Cq) value of Cq < 40.

### 2.4. Microbiological Purity

To ensure product safety, the microbiological purity of the probiotic preparations was evaluated in accordance with the requirements for both DS and FSMPs [21]. In each sample, the aerobic microbial contamination count (AMCC) and the combined yeast and mould contamination count (YMCC) were determined [33]. Additionally, to rule out the absence of potential pathogenic bacteria such as *E. coli*, *S. aureus*, *Salmonella* spp., *L. monocytogenes*, and bile-resistant Gram-negative bacteria, all of them were tested in accordance with the methods described in both the European Pharmacopoeia (Ph. Eur.) [33,34] and the United States Pharmacopeia (USP) [35,36,37]. To specifically exclude the presence of *L. monocytogenes*, samples were first enriched in Half Fraser broth (Thermo Scientific Oxoid, Basingstoke, UK) and incubated at 30–35 °C for 24–48 h, followed by plating on Brilliance Listeria Agar (Thermo Scientific Oxoid, Basingstok, UK). The agar plates were incubated at 37 °C for 24 ± 2 h before a final evaluation.

### 2.5. Biostatistics and Visualisation

All statistical analyses were performed in R (version 4.5.1; R Foundation for Statistical Computing, Vienna, Austria) using RStudio (version 2025.05.1). Data visualisation was carried out with ggplot2 (v. 3.5.1) and ggh4x (v. 0.3.1). Viability loss was quantified as a log reduction, defined as the difference between the declared viable count and the experimentally measured count, both expressed as log_10_ CFU. Formally, log reduction was calculated as

$$\Delta {log}_{10}CFU={log}_{10}(\mathrm{declared}\;\mathrm{CFU})-{log}_{10}(\mathrm{measured}\;\mathrm{CFU})$$

For each microbial species, descriptive statistics (mean, median, standard deviation, interquartile range, and sample size) were computed. Declared viable counts were treated as fixed reference values provided by manufacturers, without associated measures of variability; therefore, no formal statistical hypothesis testing between declared and observed counts was performed. Because the dataset contained small and uneven group sizes, no formal inferential statistical tests (e.g., *t*-tests or ANOVA) were performed, as such analyses would be statistically underpowered and potentially misleading. Instead, trends in viability loss as a function of the remaining shelf life (months to expiry) were explored using robust linear regression (rlm; MASS package), applied only to taxa represented by at least three observations. Robust regression was selected due to its reduced sensitivity to outliers and heteroscedasticity.

The results of these models, together with descriptive summaries, were visualised as faceted scatterplots with species-specific trend lines. Only species with at least two observations were included in the figure to ensure interpretable visualisation.

## 3. Results

### 3.1. Determination of Microorganism Count

#### 3.1.1. Pour Plate Method

The microorganism count obtained using the pour plate method (the test was performed twice, one for all products and once again for the products selected for cytometric analysis) is presented in Table 3.

A comparison of the number of probiotic microorganisms declared by manufacturers with the amount already determined experimentally in the first test showed significant differences between the analysed products. Only a few products showed a viable bacteria count close to the declared values (Crohnax IBS, Neuro LPC, Probio Slimit, Sanprobi IBS, Sanprobi Stress, Tributron), and in one case, the numbers were slightly lower but still comparable (Neurax Biotic Spectrum). However, in the majority of tested products, the number of viable microorganisms was significantly lower than the declared amount (Latopic, Biotilac IBSin, Compli Flora, Psychobiotyk CBD, Psychobiotyk IBS, SanBiotics IBS, SanBiotics Stress, Tribio DR). In the second test, all studied products except Sanprobi IBS showed a decrease in the number of viable microorganisms compared to the first test.

#### 3.1.2. Flow Cytometry

**Figure 1:**
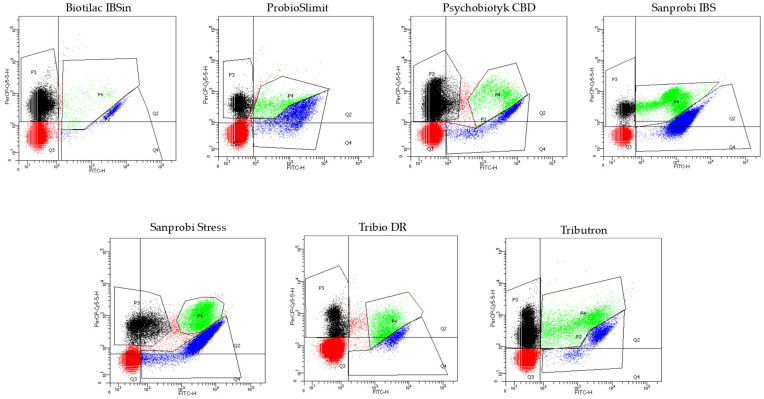
Representative multiparameter dot plots cytograms. The cells stained with cFDA (viable bacteria cells) are gated on the dot plots with blue and the cells stained with PI (non-viable bacteria cells) are gated with black. Events marked in green represent cells stained both with cFDA and PI, while those marked in red illustrate unstained cells and chemical molecules.

The seven selected probiotic products were evaluated for bacterial cell count using flow cytometry. This method revealed the presence of three distinct bacterial cell populations, namely viable cells, non-viable cells, and metabolically active cells with damaged cell walls. The results were visualised on multiparameter dot plot cytograms (Figure 1).

The number of viable and non-viable bacterial cells was calculated in accordance with ISO 19344:2015 [24]. The microorganism counts measured by flow cytometry are shown in Table 4.

The lowest number of viable bacterial cells was identified for Tribio Dr and Biotilac IBSin (5.2 × 10^8^ and 7.0 × 10^8^ AFU/dose form, respectively), and the highest was for Sanprobi Stress and Sanprobi IBS (3.6 × 10^10^ and 7.4 × 10^10^ AFU/dose form, respectively). In turn, the count of non-viable bacterial cells ranged from 1.8 × 10^9^ N-AFU/dosage form to 5.8 × 10^10^ N-AFU/dosage form.

**Figure 2:**
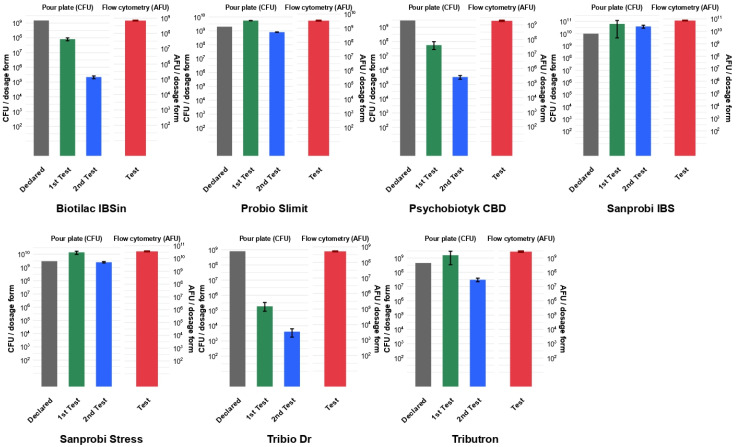
Compilation of the declared numbers of microorganisms with the results obtained using the pour plate method (performed twice at different time points) and flow cytometry.

A compilation of the declared number of microorganisms with those obtained in two pour plate tests and flow cytometry is shown in Figure 2.

Out of the seven probiotic products tested in two pour plate tests, three preparations, Probio Slimit, Sanprobi IBS, and Sanprobi Stress, obtained bacterial counts close to the declared values. In addition, they also obtained the highest values for the number of viable bacterial cells in the flow cytometry assays. For Sanprobi IBS, the declared number of bacteria was 1 × 10^10^ in both CFU and AFU/dosage form, which was consistent with the result obtained in the study.

### 3.2. Strain Identification

The results of identification using both methods, MALDI-TOF MS and qPCR, are presented in Table 5.

Due to the use of selected colonies of microorganisms obtained by the pour plate method for the identification of probiotic bacteria with MALDI-TOF MS, not all species present in some products were tested. For most isolate identifications, an SV ≥ 2.0 was achieved, indicating reliable classification at the species level. Only in one product, for the *L. helveticus* strain present in the Sanprobi Stress, an SV = 1.99 was obtained, which ensures correct identification at the genus level. Regarding the detection of yeasts in the Compli Flora and Psychobiotyk IBS formulations, MALDI-TOF MS identified them at the species level (without varieties).

In the qPCR analyses, only probiotic bacteria were subjected to identification. Reactions for *L. casei*, *B. coagulans*, and *L. delbrueckii* ssp. *bulgaricus* were not performed; therefore, these species were not analysed in Latopic, Psychobiotyk IBS, and Tribio Dr. All other declared species were detected in the study probiotics, and the Cq values obtained in the reactions were comparable to those achieved in the control samples.

### 3.3. Microbiological Purity

The results of the microbiological purity tests are shown in Table 6.

Due to the species composition of probiotic microorganisms in the product Psychobiotyk IBS, the AMCC and the YMCC were not assessed. Similarly, in the product Compli Flora, the YMCC was not determined. For eight products, the AMCCs were ≤1 × 10^1^ CFU/g, whereas for Biotilac IBSin, Crohnax IBS, SanBiotics IBS, and Sanprobi Stress, these numbers were 2.4 × 10^2^, 1.7 × 10^3^, 1.3 × 10^3^, and 2.4 × 10^4^ CFU/g, respectively. In contrast, the YMCC values for ten products were ≤1 × 10^1^ CFU/g, while for SanBiotics IBS, SanBiotics Stress, and Sanprobi Stress, these were 7.1 × 10^4^, 1.0 × 10^4^, and 6.5 × 10^1^ CFU/g, respectively. None of the tested probiotic products contained *E. coli*, *S. aureus*, *Salmonella* spp., *Listeria* spp., or bile-resistant Gram-negative bacteria in one gram of the tested sample. On the other hand, several non-declared but non-pathogenic microorganisms were detected, including *L. reuteri*, *B. animalis* ssp. *lactis*, *S. odontolytica*, *S. cerevisiae*, *S. paucimobilis*, *B. pumilus*, and *L. plantarum* in Compli Flora, Psychobiotyk CBD, Psychobiotyk IBS, SanBiotics Stress, Sanprobi Stress, and Tribio Dr, respectively.

**Figure 3:**
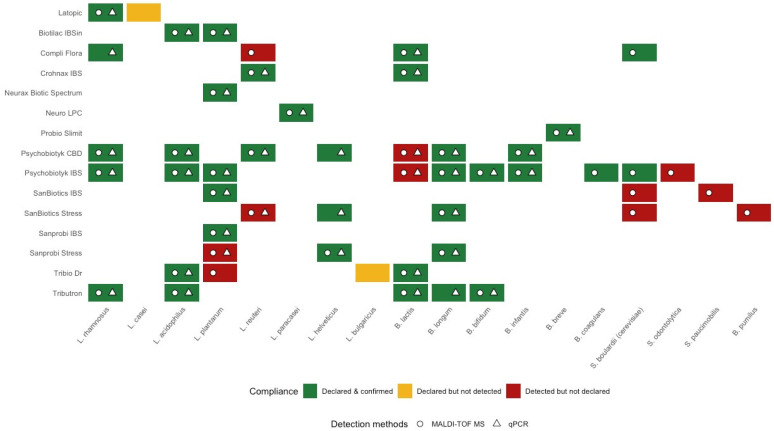
Compliance of declared and detected microorganisms.

A compliance map of declared and all detected microorganisms in tested probiotic products is shown in Figure 3.

Thirty-eight consistent (declared and confirmed) results of microorganism identification were obtained in the tested products. The vast majority of them (*n* = 31) were performed with both methods (MALDI-TOF MS and qPCR), four using only qPCR (*L. rhamnosus* in Compli Flora, *L. helveticus* in Psychobiotyk CBD and SanBiotics Stress, and *B. longum* in Tributron) and three by MALDI-TOF MS only (*B. coagulans* in Psychobiotyk IBS and *S. cerevisiae* in SanBiotics IBS and SanBiotics Stress). In two formulations, the declared microorganisms could not be identified due to limitations of the applied methods (lack of qPCR reaction primers and bacterial isolation to MALDI-TOF MS for *L. casei* in Latopic and *L. delbrueckii* ssp. *bulgaricus* in Tribio Dr). Additionally, eleven detections of unlabelled microorganisms were carried out, four using both identification methods (*L. plantarum* in Sanprobi Stress, *L. reuteri* in SanBiotics Stress, and *B. animalis* ssp. *lactis* in Psychobiotyk CBD and Psychobiotyk IBS) and seven using only MALDI-TOF MS (*L. plantarum* in Tribio Dr, *L. reuteri* in Compli Flora, *B. pumilus* in SanBiotics Stress, *S. odontolytica* in Psychobiotyk IBS, *S. paucimobilis* in SanBiotics IBS, and two *S. cerevisiae* detected in SanBiotics IBS and SanBiotics Stress preparations, where yeast should not be present).

### 3.4. Bacterial Cell Viability Assessment

Bacterial cell viability was assessed using data from studies of probiotic products exclusively containing a single species representative of the respective microbial genus. Analysis of log reductions (Δlog_10_ CFU) revealed species-specific variability in viability during storage.

The magnitude of viability loss ranged from 0 to 8 log units. Descriptive statistics showed that *L. plantarum* (*n* = 4) exhibited modest and relatively consistent reductions (median: 0.5 log), whereas *L. helveticus* (*n* = 3) and *B. longum* (*n* = 3) displayed much larger within-species variability, including individual cases of severe decline (up to 7–8 logs). In turn, yeast (*S. boulardii*, *n* = 2) demonstrated moderate and stable reductions (1–2 logs), whereas *B. breve* showed a minimal decline.

Robust linear regression models fitted for species with at least three observations indicated negative slopes for *B. longum* (−0.279 log/month), *L. helveticus* (−0.203 log/month), and *L. plantarum* (−0.060 log/month), suggesting a tendency toward an increasing loss of viable cells as products approached expiry. In contrast, *B. lactis* exhibited no clear temporal trend (+0.042 log/month).

## 4. Discussion

Research on probiotic products should focus both on their potential health-promoting properties and on assessing their quality, as these two aspects are closely related. The observed variability in viable cell counts and the presence of specific species have a direct impact on the expected physiological benefits of probiotic consumption. Products containing significantly fewer or more live microorganisms than declared may show reduced effectiveness in modulating the gut microbiota or disrupting its natural balance. In some patients, especially those with immune disorders and neonates, an excess of probiotic microorganisms can lead to their translocation from the gastrointestinal tract to other sites (e.g., the blood), causing infection [38,39,40,41].

The selection of current methods that are sensitive in detecting and identifying microorganisms is essential for robust quality control of probiotic products. In our study, we used an integrated analytical approach combining culture-dependent and culture-independent methods to evaluate the quantitative and qualitative characteristics of selected probiotics available in Polish pharmacies and to compare the obtained results with the information provided on the product labels. The advantages and limitations of the applied methods are presented in Table 7.

To maintain the declared number of viable cells until the end of the product’s shelf life, manufacturers use various strategies, including technologies slowing the release of probiotic strains, thus protecting microorganisms from gastric acid and other factors and allowing them to reach the gut, as well as controlling product-related factors such as matrix composition, packaging, water activity, temperature, and storage conditions [17]. The labels of only three tested products, Biotilac IBSin, Compli Flora, and Tributron, provided information on cell protection technologies, such as microencapsulation, extended release, and cryoprotection, respectively. The examined probiotics had the number of viable bacteria expressed in CFUs on their labels, and three of them were also in AFUs. It should be noted that all performed probiotic tests were conducted within their shelf life. The applied plate count method showed significant differences in the number of viable cells between individual products. These differences most likely reflect variation between individual commercial products, as also indicated by our log-reduction analysis, which showed high product-to-product variability and no consistent species-level patterns. Seven preparations were consistent or very close to the information provided by the manufacturer, while eight showed lower CFU values than declared. Interestingly, the latter included Biotilac IBSin and Compli Flora which, as mentioned earlier, used cell-protecting technologies. This observation is consistent with our species-level viability analysis, where the variability within individual species was large and no clear inter-species trends were observed.

Previous studies of probiotic products available on the Polish market have already shown significant discrepancies between the declared and real CFU values. A report from 2016 evaluating 25 formulations found that only one medicinal product, two DSs, and two FSMPs had viable cell counts consistent with their claimed counts [15]. Korona-Głowniak et al. [42] also confirmed the low quality of DSs and FSMPs, with only five out of ten products tested meeting the quantitative requirements for bacterial count. The issue of probiotic quality and strain viability is not limited to the Polish market. A review by Mazzantini et al. [14] presents an overall assessment of the microbiological quality of over 200 probiotics sold worldwide. Among the products for which the total CFU count was reported, more than 40% contained fewer viable cells than stated. Notably, the most frequent inconsistencies occurred in DSs and FSMPs, whereas medicinal products demonstrated higher conformity with label claims. Interestingly, a study of 12 probiotic formulations available in the United Kingdom (seven for poultry and five for humans) showed that most animal-targeted products contained counts consistent with or higher than those declared, whereas four out of five human probiotics had lower viable counts, including one product in which no viable bacteria were detected. The authors suggested that these differences likely reflected the use of spore-forming or microencapsulated strains in animal formulations, which are more tolerant to processing and storage conditions [43]. In contrast to the results presented above, a study conducted by Ghelardi et al. [16] showed that among 21 leading probiotic products marketed worldwide, the vast majority were consistent with their labels in terms of the number of viable microorganisms. Another study indicated that out of 26 probiotics sold in Slovenia, only 3 did not contain the declared number of viable microorganisms [27]. The observed differences in reports are likely due to limitations of the plate count method. Despite its technical simplicity, the method is labour-intensive and time-consuming, and the results are characterised by high variability (reaching up to 30–35%), mainly due to cell aggregation, dilution errors, and differences in media composition or incubation conditions. Furthermore, the selectivity of culture media may hinder the growth of certain strains, especially in multi-strain formulations. However, the most critical limitation is the underestimation of viable cell counts, as this method only enumerates cells capable of forming colonies under the applied growth conditions (medium, temperature, incubation time, and oxygen viability) and does not account for VBNC forms, which are unable to grow on solid media [44,45].

An alternative to the conventional plate count method is flow cytometry, which currently represents one of the most precise tools for assessing the viability of probiotic microorganisms. Flow cytometry has been shown to provide higher precision and recovery of viable cells than the plate count method, both in pure cultures and in multi-strain preparations, largely due to its ability to form VBNC bacteria [23]. Out of seven products tested by flow cytometry, only Sanprobi IBS contained the number of viable bacteria stated on the label, not only in CFUs but also in AFUs, and these values were equal. Similarly to the plate count test, where the determined CFU value was consistent with the declared one, in the flow cytometry assay, the achieved AFU value was also consistent with the claim for this supplement. Due to the fact that the six remaining products did not have the declared number of viable bacteria expressed in AFUs but only in CFUs, the results obtained from flow cytometry should not be compared with those on the labels. However, all of them obtained AFU values from flow cytometry consistent with or close to the values declared in CFUs. The correlation between AFUs and CFUs in fresh probiotic products was studied by Sielatycka et al. [46], and it was close to 1:1. In the presented study, all probiotics examined by flow cytometry were within their shelf life but at least 14 months post-manufacture. Comparing the values obtained using flow cytometry with those achieved by the simultaneously performed plate count method, significantly lower CFU values were found for the three tested products (Biotilac IBSin, Psychobiotyk CBD, and Tribio Dr). Furthermore, these values were also lower than those obtained using the previously carried out plate count method. In turn, Probio Slimit and Sanprobi Stress showed comparable AFU and CFU values obtained in both rounds of plate count methods. Finally, Tributron demonstrated consistent AFU and CFU values in the first plate count method, with a slightly lower CFU value in the second test. Overall, the best technological stability (manifested by good cultivability) was seen in products where the declared CFU values were comparable to the CFU values obtained in both rounds of the plate count method and AFU values in flow cytometry. In the probiotic that only showed consistent results with the declared CFU value in the first plate count method and AFU value in flow cytometry, the ability of bacterial cells to form colonies on a solid medium decreased over time. It should be noted that in multi-strain formulations, flow cytometry can occasionally overestimate the number of viable cells due to overlap between viable and non-viable populations on fluorescence plots for certain strains [23]. In turn, the products with the lowest culture stability were those for which the CFU values determined in the first round of the plate count method were already lower than the declared values and decreased further in the second round.

The differences in the number of microorganisms obtained by the plate count method and flow cytometry are primarily due to the fact that the former approach allows for the enumeration of bacteria only capable of replicating in a suitable environment but not metabolically active cells (VBNCs) that are unable to grow on solid media [47]. In addition, discrepancies between CFU and AFU values may be particularly evident in probiotic preparations containing multiple strains, where microbial interactions such as competition for nutrients or production of inhibitory metabolites may inhibit the growth of certain strains on solid media [46]. The final microorganism count result is also significantly influenced by incubation parameters such as time, temperature, and oxygen availability, which must be optimised for each bacterial species [48]. However, it should be noted that although the CFU values for some tested supplements were lower than expected, all had high AFU values and, therefore, their beneficial properties in the indicated physiological disorders were most likely preserved, as the VBNCs present in all formulations can regain metabolic activity and growth capacity under favourable conditions, such as those found in the human gastrointestinal tract [49,50,51].

The accurate identification of microorganisms is a key aspect of assessing the quality and reliability of commercial probiotic products. In recent years, MALDI-TOF MS has become one of the most important tools for rapid microbial detection, although the application of this technique for the identification of certain species remains challenging due to subtle differences between strains that are often undetectable in mass spectrometry analyses [26,27]. Furthermore, the precision of this instrument depends largely on the quality and continuous updating of the reference databases used for spectrum comparison [52]. All morphologically distinct microbial colonies obtained in the first round of the plate count method were identified by MALDI-TOF MS. Detection was not performed for *L. casei* in Latopic, *L. rhamnosus* in Compli Flora, *L. helveticus* in Psychobiotic CBD, *L. helveticus* in SanBiotics Stress, *L. delbrueckii* ssp. *bulgaricus* in Tribio Dr, and *B. longum* in Tributron solely because colonies of these species were not collected for testing. All other bacteria present in the studied probiotic products were correctly identified. The detection of yeasts present in the two preparations (SanBiotics IBS and SanBiotics Stress) using MALDI-TOF MS was also accurate; however, this method yielded identification only at the species level, whereas the manufacturers reported a specific strain variant. The complementary method used to detect the declared probiotic bacteria was a culture-independent qPCR assay. Compared to the culture-based approach, qPCR is faster and allows for the detection of even small bacterial populations within dominant populations [53]. All qPCR tests carried out proved effective in identifying species. Due to the lack of starters for the identification of *L. casei*, *B. coagulans*, and *L. delbrueckii* ssp. *bulgaricus*, these microorganisms were not studied in Latopic, Psychobiotic IBS, and Tribio Dr, respectively.

The microbiological purity of probiotic products constitutes one of the key criteria for evaluating their quality and safety of use. According to the USP guidelines for dietary supplements, the total count of aerobic mesophilic bacteria should not exceed 5 × 10^3^ CFU/g [35]. One of the tested products, Sanprobi Stress, exceeded this level, achieving a result of 2.4 × 10^4^, and identification using both MALDI-TOF MS and qPCR revealed the presence of an unreported *L. plantarum* strain. It should be noted that bacteria belonging to the *Lactobacillaceae* family are facultative anaerobes and therefore can grow in an aerobic environment [54,55]. The safe limit for AMCCs was not exceeded in the other preparations, although undeclared bacterial strains were found in some of them. Contamination with the *L. reuteri* strain was detected by both MS and qPCR methods in SanBiotics Stress. Also, using both identification tests, unreported *B. animalis* spp. *lactis* was detected in two preparations, Psychobiotyk CBD and Psychobiotyk IBS. In turn, bacteria belonging to *Bifidobacterium* spp. are strict anaerobes, although *B. animalis* spp. *lactis* is the most aerotolerant species among them and therefore can grow under aerobic conditions [56]. Due to the finding that all of the above-mentioned contaminants belong to probiotic bacteria preferring anaerobic conditions, it seems appropriate to exclude them from the total AMCCs. The study also included cases where an unreported probiotic bacterial strain was identified exclusively by MALDI-TOF. This concerned *L. reuterii* and *L. plantarum* in Compli Flora and Tribio Dr, respectively. Importantly, the qPCR approaches did not confirm the presence of these species but identified the declared and simultaneously undetected-by-MS strains of *L. rhamnosus* and *L. acidophilus*, respectively. Considering that the MALDI-TOF MS detects only a fragment of the bacterial proteome within a limited mass range, it appears that this method yielded incorrect identification for closely related species of the *Lactobacillaceae* family [27]. This highlights the need to combine culture-based, proteomic, and molecular methods for robust and reliable strain-level identification. Several samples also identified aerobic non-pathogenic bacteria, such as *S. odontolytica*, *S. paucimobilis*, and *B. pumilus* in Psychobiotyk IBS, SanBiotics IBS, and SanBiotics Stress, respectively. The last two preparations also contained undeclared *S. cerevisiae* in amounts exceeding the acceptable YMCCs. Of concern is that the vast majority of contaminants were detected in products from a single manufacturer. Importantly, none of the tested probiotic products contained pathogens like, *E. coli*, *S. aureus*, *Salmonella* spp., *Listeria* spp., or bile-resistant Gram-negative bacteria. However, as emphasised by Tyski et al. [57], even non-pathogenic microorganisms present in medicinal or dietary products can negatively affect product stability and overall quality.

A comprehensive review by Mazzantini et al. [14] demonstrated that contamination of probiotic products may pose a significant problem worldwide. The authors reported the presence of potential pathogens in 22.6% of medicinal products and 16% of dietary supplements, whereas all analysed functional food products were free of contaminants. In some products, contamination levels were very high, reaching up to 10^11^ CFU per dose, and the detected microorganisms included *Bacillus cereus*, *Bacillus licheniformis*, *Bacillus thuringiensis*, *Enterococcus faecium*, *Staphylococcus epidermidis*, and *Klebsiella* spp. In contrast, Ghelardi et al. [16] evaluated probiotic products available worldwide and confirmed a high level of microbiological purity, and no undesirable microorganisms were detected in any of the tested samples.

The conducted study also attempted to assess the viability of bacterial cells. Overall, the data indicate that viability loss varied substantially between products and species. Based on robust regression applied to species with at least three observations, only *B. longum* and *L. helveticus* showed clear negative slopes, whereas *L. plantarum* exhibited only a weak decline and *B. lactis* showed no discernible temporal trend. Species represented by two data points (including *S. boulardii*) cannot be reliably evaluated. Considering the limited sample size, uneven representation of species, and product heterogeneity, these observations should be regarded as preliminary and interpreted with caution.

## 5. Limitations

The presented study has several limitations, as follows: (i) not all analysed preparations were tested by flow cytometry (restriction resulting from expiry dates; only preparations that were within their shelf life were used for the tests), (ii) not all microorganisms declared in the products were identified by MALDI-TOF MS (limitations of the culture-dependent method and the commercial data library; not all microbial colonies were selected for the study and there were difficulties in identifying closely related species from the *Lactobacillaceae* family), (iii) not all microorganisms were identified by qPCR (restriction due to the lack of primers for some species, e.g., *L. casei*, *B. coagulans*, and *L. delbrueckii* ssp. *bulgaricus*), and (iv) the number of observations per species was insufficient to reliably evaluate viability trends over storage time.

## 6. Conclusions

The efficacy of probiotic supplementation depends primarily on the delivery of an adequate number of viable microorganisms capable of surviving gastrointestinal transit and contributing to gut colonisation. Insufficient CFU levels may substantially limit the expected health benefits, particularly in patients with gastrointestinal disorders such as IBS, allergies, or depression, where effective restoration of microbial balance is crucial for therapeutic outcomes. Similarly, in immunocompromised individuals, low viability of probiotic strains may result in inadequate immune support. While reduced CFU concentrations are unlikely to pose a risk to healthy consumers, they may diminish or completely negate the potential benefits associated with maintaining gut homeostasis or modulating the intestinal microbiota. In this context, this study conducted a comprehensive assessment of the microbiological quality, strain composition, and viability of selected probiotic products available on the Polish market. It was shown that the quality and biological consistency of commercial products may differ significantly from the declared specifications, which may affect their clinical and functional efficacy. The results of the study highlight the importance of implementing standardised analytical methods and harmonised quality control procedures for probiotic products. In addition, consumer education initiatives and independent testing programmes can play a valuable role in supporting informed product choices and increasing public confidence in probiotic supplementation.

## Figures and Tables

**Table 1:** Characteristics of tested probiotic products.

Probiotic Product (Manufacturer)	Dosage Forms	Indication	Temperature of Storage: R/F
**Latopic** (IBSS BIOMED, Krakow, Poland)	Capsules	food allergy, AD	R
**Biotilac IBSin** (COLFARM, Mielec, Poland)	Capsules	IBS	R
**Compli Flora** (Pamex Pharmaceuticals, Selters, Germany)	Capsules	diarrhoea, food allergies, during/after antibiotic therapy	R
**Crohnax IBS** (Farmina, Krakow, Poland)	Capsules	restoration of gut microbiota, bloating symptoms, abdominal pain, obstipation	F
**Neurax Biotic Spectrum** (Bened Biomedical Co., Istrana, Italy)	Sachets	autism spectrum disorders (alleviating aggression, anxiety, rule-breaking, hyperactivity)	R
**Neuro LPC** (Kosma Pharma Group, Warsaw, Poland)	Capsules	restoration of gut microbiota, stress, low mood	R
**Probio Slimit** (Aflofarm Farmacja Polska, Pabianice, Poland)	Capsules	restoration of gut microbiota, fat burning, weight control	R
**Psychobiotyk CBD** (UNIPRO, Kłaj, Poland)	Capsules	restoration of gut microbiota, support for mental/physical health	R
**Psychobiotyk IBS** (UNIPRO, Kłaj, Poland)	Capsules	fatigue, depression, IBS	R
**SanBiotics IBS** (UNIPRO, Kłaj, Poland)	Capsules	IBS	R
**SanBiotics Stress** (UNIPRO, Kłaj, Poland)	Capsules	nervous tension, malaise, improved digestion, restoration of gut microbiota	R
**Sanprobi IBS** (Sanprobi, Szczecin, Poland)	Capsules	abdominal pain, constipation, bloating, restoration of gut microbiota	R
**Sanprobi Stress** (Sanprobi, Szczecin, Poland)	Capsules	restoration of gut microbiota, stress	R
**Tribio Dr** (DIATHER Petrusewicz, Gdańsk, Poland)	Capsules	restoration of gut microbiota, during/after antibiotic therapy, AD, allergies	R
**Tributron** (Aurovitas Pharma Polska, Warsaw, Poland)	Capsules	restoration of gut microbiota, during/after antibiotic therapy, IBS	R

Abbreviations: AD—atopic dermatitis; IBS—irritable bowel syndrome; R—room temperature (15–25 °C); F—refrigerator temperature (2–8 °C).

**Table 2:** Primers used for qPCR strain identification.

Species	Target Gene/Region	Primer Name	Sequence (5′-3′)	References
*L. rhamnosus*	16S–23S	Rhamnosus-F	GCC GAT CGT TGA CGT TAG TTG G	[29]
		Rhamnosus-R	CAG CGG TTA TGC GAT GCG AAT	
*L. paracasei*	Cation transport ATPase	Paracasei-F	CAA TGC CGT GGT TGT TGG AA	[29]
		Paracasei-R	GCC AAT CAC CGC ATT AAT CG	
*L. acidophilus*	16S–23S	Acidophilus-F	CCT TTC TAA GGA AGC GAA GGA T	[29]
		Acidophilus-R	ACG CTT GGT ATT CCA AAT CGC	
*L. plantarum*	LPXTG motif cell wall anchor domain protein	Plantarum-F	GCT GGC AAT GCC ATC GTG CT	[29]
		Plantarum-R	TCT CAA CGG TTG CTG TAT CG	
*L. helveticus*	ACPS-malonyltransferase	Helveticus-F	GTA TGA TCG TTC GCC ACC AC	[30]
		Helveticus-R	ATT GTC GCC ATG AGT ACA GG	
*L. reuteri*	16S–23S	Reuteri-F	GAT TGA CGA TGG ATC ACC AGT	[29]
		Reuteri-R	CAT CCC AGA GTG ATA GCC AA	
*B. bifidium*	16S–23S	Bifidium-F	CCA CAT GAT CGC ATG TGA TTG	[31]
		Bifidium-R	CCG AAG GCT TGC TCC CAA A	
*B. breve*	16S–23S	Breve-F	CCG GAT CGT CCA TCA CAC	[32]
		Breve-R	ACA AAG TGC CTT GCT CCC T	
*B. longum*	16S–23S	Longum-F	TTC CAG TTG ATC GCA TGG TC	[31]
		Longum-R	GGG AAG CCG TAT CTC TAC GA	
*B. lactis*	16S–23S	Lactis-F	ACC TCA CCA ATC CGC TGT TC	[32]
		Lactis-R	GAT CCG CAT GGT GGA ACT CT	
*B. infantis*	16S–23S	Infantis-F	TTC CAG TTG ATC GCA TGG TC	[31]
		Infantis-R	GGA AAC CCC ATC TCT GGG AT	

**Table 3:** Number of declared probiotic microorganisms determined by the pour plate method.

Probiotic Product	Microorganism	Declared CFU/Dosage Form	1st Test		2nd Test	
			Exp. Date (Month)	Obtained CFU/Dosage Form (Confidence Interval)	Exp. Date (Month)	Obtained CFU/Dosage Form (Confidence Interval)
Latopic	*Lactobacillaceae*	1.5 × 10^9^	9	9.3 × 10^1^ (8.1 × 10^1^–1.1 × 10^2^)	nt	nt
Biotilac IBSin	*Lactobacillaceae*	1 × 10^10^	19	8.1 × 10^7^ (6.3 × 10^7^–9.9 × 10^7^)	5	2.2 × 10^5^ (1.7 × 10^5^–2.6 × 10^5^)
Compli Flora	*Lactobacillaceae*	2 × 10^9^	2	4.6 × 10^5^ (4.1 × 10^5^–5.1 × 10^5^)	nt	nt
	*Bifidobacterium* spp.	2 × 10^9^		3.6 × 10^7^ (3.2 × 10^7^–3.9 × 10^7^)	nt	nt
	*Saccharomyces* spp.	2 × 10^9^		4.7 × 10^7^ (4.6 × 10^7^–4.7 × 10^7^)	nt	nt
**Crohnax IBS**	*Bifidobacterium* spp.	Total 5 × 10^9^	18	7.2 × 10^9^ (5.4 × 10^9^–9.1 × 10^9^)	nt	nt
	*Lactobacillaceae*			5.4 × 10^9^ (5.0 × 10^9^–5.7 × 10^9^)	nt	nt
Neurax Biotic Spectrum	*Lactobacillaceae*	3 × 10^10^	9	6.7 × 10^9^ (5.4 × 10^9^–8.0 × 10^9^)	nt	nt
**Neuro LPC**	*Lactobacillaceae*	2.5 × 10^10^	23	4.1 × 10^10^ (3.8 × 10^10^–4.3 × 10^10^)	nt	nt
**Probio Slimit**	*Bifidobacterium* spp.	2 × 10^9^	16	5.5 × 10^9^ (5.3 × 10^9^–5.6 × 10^9^)	2	8.1 × 10^8^ (7.8 × 10^8^–8.4 × 10^8^)
Psychobiotyk CBD	*Bifidobacterium* spp.	2 × 10^9^	16	7.9 × 10^7^ (6.1 × 10^7^–9.8 × 10^7^)	3	3.7 × 10^5^ (3.1 × 10^5^–4.2 × 10^5^)
	*Lactobacillaceae*	4 × 10^9^		3.4 × 10^7^ (2.8 × 10^7^–4.0 × 10^7^)		3.0 × 10^5^ (2.3 × 10^5^–3.6 × 10^5^)
Psychobiotyk IBS	*Bacillus* spp.	1 × 10^8^	11	5.6 × 10^6^ (4.9 × 10^6^–6.3 × 10^6^)	nt	nt
	*Bifidobacterium* spp.	3 × 10^9^		8.2 × 10^7^ (6.1 × 10^7^–1.0 × 10^8^)	nt	nt
	*Lactobacillaceae*	3 × 10^9^		5.1 × 10^7^ (5.0 × 10^7^–5.3 × 10^7^)	nt	nt
	*Saccharomyces* spp.	1 × 10^9^		7.3 × 10^8^ (5.8 × 10^8^–8.9 × 10^8^)	nt	nt
SanBiotics IBS	*Lactobacillaceae*	1 × 10^10^	10	9.4 × 10^7^ (8.6 × 10^7^–1.0 × 10^8^)	nt	nt
SanBiotics Stress	*Bifidobacterium* spp.	1.6 × 10^9^	8	2.5 × 10^1^ (1.6 × 10^1^–3.5 × 10^1^)	nt	nt
	*Lactobacillaceae*	1.4 × 10^9^		4.3 × 10^3^ (3.9 × 10^3^–4.7 × 10^3^)	nt	nt
**Sanprobi IBS**	*Lactobacillaceae*	1 × 10^10^	22	6.3 × 10^10^ (4.5 × 10^9^–1.2 × 10^11^)	8	3.9 × 10^10^ (2.8 × 10^10^–4.9 × 10^10^)
**Sanprobi Stress**	*Lactobacillaceae*	Total 3 × 10^9^	20	1.4 × 10^10^ (1.2 × 10^10^–1.7 × 10^10^)	6	2.5 × 10^9^ (2.2 × 10^9^–2.8 × 10^9^)
	*Bifidobacterium* spp.			1.0 × 10^9^ (9.0 × 10^8^–1.2 × 10^9^)		4.3 × 10^8^ (3.9 × 10^8^–4.7 × 10^8^)
Tribio Dr	*Lactobacillaceae*	9 × 10^8^	23	8.2 × 10^3^ (6.9 × 10^3^–9. × 10^3^)	11	6.0 × 10^3^ (5.7 × 10^3^–6.3 × 10^3^)
	*Bifidobacterium* spp.	7 × 10^8^		3.8 × 10^5^ (3.4 × 10^5^–4.3 × 10^5^)		2.4 × 10^3^ (1.8 × 10^3^–3.0 × 10^3^)
**Tributron**	*Bifidobacterium* spp.	7.2 × 10^8^	18	2.6 × 10^9^ (2.3 × 10^9^–3.0 × 10^9^)	4	2.7 × 10^3^ (2.1 × 10^3^–3.3 × 10^3^)
	*Lactobacillaceae*	2.8 × 10^8^		1.3 × 10^8^ (1.1 × 10^8^–1.4 × 10^8^)		3.0 × 10^7^ (2.2 × 10^7^–3.9 × 10^7^)

Abbreviations: CFU—colony forming unit; Exp. date—expiration date; nt—not tested. The names of products in which the number of bacteria was consistent with the manufacturer’s declaration are highlighted in bold.

**Table 4:** Number of probiotic bacteria determined by flow cytometry.

Probiotic Product	Microorganism	AFU/Dosage Form (Confidence Interval)	N-AFU/Dosage Form (Confidence Interval)
Biotilac IBSin	*Lactobacillaceae*	7.0 × 10^8^ (6.9 × 10^8^–7.1 × 10^8^)	3.6 × 10^10^ (3.3 × 10^10^–3.9 × 10^10^)
Probio Slimit	*Bifidobacterium* spp.	3.4 × 10^9^ (3.3 × 10^9^–3.5 × 10^9^)	7.0 × 10^9^ (6.9 × 10^9^–7.1 × 10^9^)
Psychobiotyk CBD	*Bifidobacterium* spp. *Lactobacillaceae*	1.9 × 10^9^ (1.8 × 10^9^–2.0 × 10^9^)	2.1 × 10^10^ (2.0 × 10^10^–2.2 × 10^10^)
Sanprobi IBS	*Lactobacillaceae*	7.4 × 10^10^ (7.3 × 10^10^–7.5 × 10^10^)	6.2 × 10^9^ (6.1 × 10^10^–6.3 × 10^10^)
Sanprobi Stress	*Lactobacillaceae* *Bifidobacterium* spp.	3.6 × 10^10^ (3.5 × 10^10^–3.7 × 10^10^)	5.8 × 10^10^ (5.7 × 10^10^–5.9 × 10^10^)
Tribio Dr	*Lactobacillaceae* *Bifidobacterium* spp.	5.2 × 10^8^ (5.1 × 10^8^–5.3 × 10^8^)	1.8 × 10^9^ (1.6 × 10^9^–2.0 × 10^9^)
Tributron	*Bifidobacterium* spp. *Lactobacillaceae*	2.8 × 10^9^ (2.6 × 10^9^–3.0 × 10^9^)	2.5 × 10^10^ (2.3 × 10^10^–2.7 × 10^10^)

Abbreviations: AFU—active fluorescence unit; N-AFU—nonactive fluorescence unit.

**Table 5:** Microorganism identification obtained by MALDI-TOF MS and qPCR methods.

Probiotic Product	Declared Strain	MALDI-TOF MS		qPCR		
		Identification	SV	Identification	Test Sample Cq	Positive Control Cq
Latopic	*L. rhamnosus* ŁOCK 0908	*L. rhamnosus*	2.25	*L. rhamnosus*	15	16
	*L. rhamnosus* ŁOCK 0900					
	*L. casei* ŁOCK0919	nd	-	nt	-	-
Biotilac IBSin	*L. acidophilus* LA02	*L. acidophilus*	2.45	*L. acidophilus*	24	19
	*L. plantarum* LP01	*L. plantarum*	2.47	*L. plantarum*	19	17
Compli Flora	*L. rhamnosus* GG	nd	-	*L. rhamnosus*	19	16
	*B. lactis* BS01	*B. animalis* ssp. *lactis*	2.63	*B. lactis*	23	18
	*S. boulardii*	*S. cerevisiae*	2.13	nt	-	-
Crohnax IBS	*B. lactis* 5764	*B. animalis* ssp. *lactis*	2.59	*B. lactis*	17	18
	*L. reuteri* 5454	*L. reuteri*	2.18	*L. reuteri*	15	12
Neurax Biotic Spectrum	*L. plantarum* PS128	*L. plantarum*	2.32	*L. plantarum*	17	17
Neuro LPC	*L. paracasei* (Lpc-37)	*L. paracasei* ssp. *paracasei*	2.37	*L. paracasei*	14	16
Probio Slimit	*B. breve* B-3	*B. breve*	2.34	*B. breve*	18	25
Psychobiotyk CBD	*B. infantis* BI02	*B. longum* ssp. *infantis*	2.41	*B. infantis*	16	25
	*B. longum* BL-G301	*B. longum*	2.35	*B. longum*	34	33
	*L. acidophilus* LA02	*L. acidophilus*	2.47	*L. acidophilus*	21	19
	*L. reuteri* LRE02	*L. reuteri*	2.31	*L. reuteri*	16	12
	*L. rhamnosus* GG	*L. rhamnosus*	2.37	*L. rhamnosus*	18	16
	*L. helveticus* CNCMI-3360	nd	-	*L. helveticus*	19	16
Psychobiotyk IBS	*B. coagulans*	*B. coagulans*	2.15	nt	-	-
	*B. bifidum*	*B. bifidum*	2.37	*B. bifidum*	20	25
	*B. infantis*	*B. longum* ssp. *infantis*	2.07	*B. infantis*	17	25
	*B. longum*	*B. longum*	2.14	B. longum	35	33
	*L. acidophilus*	*L. acidophilus*	2.30	L. acidophilus	21	19
	*L. plantarum*	*L. plantarum*	2.38	*L. plantarum*	21	17
	*L. rhamnosus*	*L. rhamnosus*	2.32	*L. rhamnosus*	19	16
	*S. boulardii*	*S. cerevisiae*	2.05	nt	-	-
SanBiotics IBS	*L. plantarum* LP09 DSM 25710	*L. plantarum*	2.41	*L. plantarum*	21	17
SanBiotics Stress	*B. longum* CNCM I-5097	*B. longum*	2.31	*B. longum*	38	32
	*L. helveticus* LMG P-31392	nd	-	*L. helveticus*	19	16
Sanprobi IBS	*L. plantarum* 299v	*L. plantarum*	2.48	*L. plantarum*	17	17
Sanprobi Stress	*L. helveticus* Rosell-2	*L. helveticus*	1.99	*L. helveticus*	17	16
	*B. longum* Rosell-175	*B. longum*	2.25	*B. longum*	36	33
Tribio Dr	*L. acidophilus*	*L. acidophilus*	2.58	*L. acidophilus*	21	19
	*L. delbrueckii* ssp. *bulgaricus*	nd	-	nt	-	-
	*B. lactis*	*B. animalis* ssp. *lactis*	2.44	*B. lactis*	24	18
Tributron	*B. lactis* Flora Active 32269	*B. animalis* ssp. *lactis*	2.45	*B. lactis*	19	18
	*B. bifidum* Flora Active 32403	*B. bifidum*	2.36	*B. bifidum*	19	24
	*B. longum* Flora Active 32946	nd	-	*B. longum*	33	33
	*L. rhamnosus* Flora Active 19070-2	*L. rhamnosus*	2.36	*L. rhamnosus*	17	16
	*L. acidophilus* Flora Active 32418	*L. acidophilus*	2.32	*L. acidophilus*	20	19

Abbreviations: nd—not done; nt—not tested; SV—score value; Cq—cycle quantification value.

**Table 6:** Microbiological purity results.

Probiotic Product	AMCC [CFU/g]	YMCC [CFU/g]	*E. coli* (1 g)	*S. aureus* (1 g)	*Salmonella* spp. (1 g)	*Listeria* spp. (1 g)	Gram-Negative Bile-Tolerant Bacteria (1 g)	Other Contaminants
Latopic	<1 × 10^1^	<1 × 10^1^	nd	nd	nd	nd	nd	nd
Biotilac IBSin	2.4 × 10^2^	<1 × 10^1^	nd	nd	nd	nd	nd	nd
Compli Flora	<1 × 10^1^	nt	nd	nd	nd	nd	nd	*L. reuteri*
Crohnax IBS	1.7 × 10^3^	<1 × 10^1^	nd	nd	nd	nd	nd	nd
Neurax Biotic Spectrum	<1 × 10^1^	<1 × 10^1^	nd	nd	nd	nd	nd	nd
Neuro LPC	<1 × 10^1^	<1 × 10^1^	nd	nd	nd	nd	nd	nd
Probio Slimit	3.5 × 10^1^	<1 × 10^1^	nd	nd	nd	nd	nd	nd
Psychobiotyk CBD	1.0 × 10^1^	<1 × 10^1^	nd	nd	nd	nd	nd	*B. animalis* ssp. *lactis*
Psychobiotyk IBS	nt	nt	nd	nd	nd	nd	nd	*Schaalia odontolytica* *B. animalis* ssp. *lactis*
SanBiotics IBS	1.3 × 10^3^	7.1 × 10^4^	nd	nd	nd	nd	nd	*S. cerevisiae*, *Sphingomonas paucimobilis*
SanBiotics Stress	<1 × 10^1^	1.0 × 10^4^	nd	nd	nd	nd	nd	*S. cerevisiae* *Bacillus pumilus* *L. reuteri*
Sanprobi IBS	<1 × 10^1^	<1 × 10^1^	nd	nd	nd	nd	nd	nd
Sanprobi Stress	2.4 × 10^4^	6.5 × 10^1^	nd	nd	nd	nd	nd	*L. plantarum*
Tribio Dr	1.5 × 10^1^	<1 × 10^1^	nd	nd	nd	nd	nd	*L. plantarum*
Tributron	<1 × 10^1^	<1 × 10^1^	nd	nd	nd	nd	nd	nd

Abbreviations: nt—not tested; nd—not detected; AMCC—aerobic microbial contamination count; YMCC—yeast and mould contamination count.

**Table 7:** Characteristics of the research methods used in the study.

Methods	Advantages	Limitations
Pour plate method	Enables isolation of pure microbial cultures Allows for detailed analysis of colony morphology and growth characteristics Facilitates quantitative estimation of viable cells (e.g., CFU determination) Provides material for downstream analyses (e.g., biochemical, molecular, or antimicrobial susceptibility testing) High reproducibility and methodological standardisation Cost-effective and widely accessible technique	Limited to microorganisms capable of growth under laboratory conditions Time-consuming due to incubation requirements Underestimation of total microbial diversity (viable but non-culturable organisms are excluded) Selective media and growth conditions may bias community composition Risk of contamination during manual handling Slow-growing organisms may be masked by rapidly proliferating species
Flow cytometry	Ability to analyse large numbers of cells Short turnaround time for obtaining results Simultaneous assessment of multiple parameters (e.g., cell size and granularity) No requirement for cell cultivation High measurement accuracy Low risk of sample contamination Capability to enumerate viable but non-culturable (VBNC) bacteria	High cost of reagents and instrumentation Autofluorescence of certain cell types Spectral overlap when multiple fluorochromes are used Lack of straightforward identification of individual strains
qPCR	Very high specificity allowing for species-level identification without prior culture-based enrichment High analytical sensitivity, allowing for detection of a single DNA copy Rapid results (~4 h: DNA extraction and amplification) PMA/EMA-qPCR discriminates live from dead cells (99.96–100% inhibition of DNA from dead cells) The ability to simultaneously identify multiple strains using multiplex PCR High-throughput analysis of hundreds of samples simultaneously; ideal for manufacturer quality control	Requires optimisation of primers and probes for each microorganism Higher cost of equipment and analysis Potential PCR inhibitors in complex probiotic matrices (e.g., capsules, yoghurts) Does not directly measure metabolic activity (only the presence of DNA/RNA) Lack of a formal ISO standard for probiotics (in contrast to flow cytometry, ISO 19344) Inter-laboratory variability in the absence of standardised protocol
MALDI-TOF MS	Rapid and accurate identification of bacteria at the species level Allows for analysis of multiple colonies in a short time	Difficulties in distinguishing very closely related strains (e.g., closely related *Lactobacillaceae* species) Accuracy depends on the quality and currency of reference databases Expensive method and requires specialised equipment

## Data Availability

The original contributions presented in this study are included in the article. Further inquiries can be directed to the corresponding authors.

## Abbreviations

The following abbreviations are used in this manuscript:

AD	Atopic Dermatitis
AFU	Active Fluorescence Units
AMCC	Aerobic Microbial Contamination Count
ATCC	American Type Culture Collection
BSM	Bifidobacteria Selective Medium
cFDA	Fluorescent Dyes, 5-(And-6)-Carboxyfluorescein Diacetate
CFU	Colony-Forming Unit
Cq	Cycle Quantification
DSM	German Collection Of Microorganisms
DSs	Dietary Supplements
Exp. Date	Expiration Date
F	Refrigerator Temperature (2–8 °C)
FSMPs	Foods For Special Medical Purposes
IBS	Irritable Bowel Syndrome
ISO	International Standard Organization
MALDI-TOF MS	Matrix-Assisted Laser Desorption/Ionisation Time-Of-Flight Mass Spectrometry
MRS Medium	Deman Rogosa And Sharpe Medium
N-AFU	Nonactive Fluorescence Units
Nd	Not Done
Nt	Not Tested
PCR	Polymerase Chain Reaction
Ph. Eur.	European Pharmacopoeia
PI	Propidium Iodide
qPCR	Real-Time Quantitative Polymerase Chain Reaction
R	Room Temperature (15–25 °C)
SV	Score Value
USP	United States Pharmacopeia
VBNC	Viable But Non-Culturable
YMCC	Yeast And Mould Contamination Count
